# Diagnosing gastrointestinal stromal tumors: The utility of fine‐needle aspiration cytology versus biopsy

**DOI:** 10.1002/cam4.4630

**Published:** 2022-03-17

**Authors:** Yifan Zhang, Sara Renberg, Andri Papakonstantinou, Felix Haglund de Flon

**Affiliations:** ^1^ Department of Clinical Pathology and Cytology Karolinska University Hospital Stockholm Sweden; ^2^ Department of Oncology‐Pathology Karolinska Institutet Stockholm Sweden; ^3^ Department of Head, Neck, Lung and Skin Tumors Karolinska University Hospital Stockholm Sweden; ^4^ Department of Breast cancer, Endocrine tumors and Sarcoma Karolinska University Hospital Stockholm Sweden

**Keywords:** biopsy, cytology, diagnosis, GIST, mutation, sarcoma

## Abstract

**Background:**

Gastrointestinal stromal tumors (GIST) are mesenchymal tumors in the intestinal tract originating from a precursor to the interstitial cells of Cajal, which plays a role in gastric motility. The preoperative diagnosis of GIST may be very important for the surgical approach or the need for neoadjuvant treatment and is often done in conjunction with molecular testing.

**Design:**

GISTs diagnosed in Stockholm between 1999 and 2019 with biopsy and/or fine‐needle aspiration (FNA) material were included. Clinical and tumor characteristics, as well as sample representability, ancillary techniques, diagnostic accuracy, and time to diagnosis, were categorized and compared.

**Results:**

We identified 460 diagnostic samples from 347 patients, consisting of 212 biopsies and 248 FNAs. FNA cytology had a significantly (*p* < 0.05) better sample representability (92% vs. 77%), diagnostic accuracy (84% vs. 76%), and shorter time to diagnosis (4.5 vs. 12.3 days on average) in comparison with biopsies. In addition, ancillary techniques such as immunochemistry and molecular analysis for KIT and PDGFRA mutations could satisfactorily be performed on FNA materials.

**Conclusions:**

There are advantages to both biopsy and FNA cytology in diagnosing GISTs. While the significantly shorter time to diagnosis for FNA cytology can be due to institutional differences, its many strengths make it both an accurate and time‐efficient method for preoperative diagnosis of GIST.

## Graphical abstract

Preoperative diagnosis of gastrointestinal stromal tumors is important for risk assessment and eventual neoadjuvant treatment. We found fine needle aspiration to be a accurate diagnostic method, with higher time‐efficiency compared to core needle biopsy.

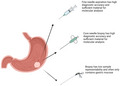

## 1 INTRODUCTION

Gastrointestinal stromal tumors (GIST) are mesenchymal tumors in the intestinal tract and are believed to originate from precursors to the interstitial cells of Cajal, which play a role in gastric motility.^1^, ^2^, ^3^ They are thought to be sporadically arising tumors, with no known risk factors and only rarely associated with tumor syndromes, such as Neurofibromatosis 1 or succinate dehydrogenase (SDH) deficiency.^4^ Although GISTs may affect patients of all age groups, they are more common in the elderly population, with a median age of diagnosis at 60 years.^5^, ^6^

Gastrointestinal stromal tumors may arise anywhere in the digestive tract but most often occur in the stomach or small intestine.^5^ Symptoms may vary depending on tumor location, but the loss of appetite and abdominal pain are common symptoms. It is not uncommon for GISTs to present with anemia due to gastrointestinal tumor bleeding or, rarely, tumor perforation and acute peritonitis.^7^

Current risk stratification is based on anatomical location, tumor size, and mitosis count per 50 high‐powered fields (HPFs).^6^ Tissue samples are generally collected via endoscopic ultrasound, either via a biopsy—core‐needle biopsies or forceps—or fine‐needle aspiration (FNA) cytology. The tumors are comprised of spindle cells or more rarely epithelioid cells and almost always show immunoreactivity for CD117 and DOG‐1.^8^

The majority of GISTs are associated with activating mutations in the KIT or PDGFRA genes, which code for receptor tyrosine kinases. The described mutations in KIT are commonly in exons 11, 9, 13, and 17.^8^, ^9^, ^10^ Mutations in PDGFRA are less common and usually in exons 12 or 18. KIT and PDGFRA mutations are mutually exclusive. A small number of GISTs are associated with other mutations, for example, in NF1 or the SDH‐gene family,^10^, ^11^ and in very rare cases, associated with familial GIST, an autosomal‐dominant genetic disorder with germline KIT mutations.^12^

An adequate preoperative diagnosis is vital for proper management and informed treatment decisions. For example, while surgical resection is the primary treatment for localized tumors, neoadjuvant Imatinib (Glivec), an inhibitor of the KIT‐receptor, may be administered for initially inoperable tumors or for downstaging purposes in tumors located at sensitive anatomical structures.^13^, ^14^, ^15^

Although GISTs are traditionally diagnosed with biopsies, FNA cytology is also an excellent method and can provide an accurate diagnosis. However, several previous studies have shown varying sample representability as well as suboptimal sample yield in FNAs compared with biopsies, often producing insufficient materials for ancillary studies.^16^, ^17^, ^18^ In this study, we systematically examined and compared diagnosed GISTs by either biopsy or FNA at Stockholm's hospitals during a 20‐year period.

## 2 MATERIALS AND METHODS

### 2.1 Patient samples

Patients diagnosed with GISTs diagnosed between 1999 and 2019 were identified by searching the digital archives of all Departments of Pathology and Cytology in Stockholm (Karolinska University Hospital Solna and Huddinge, Södersjukhuset and Danderyds Hospital). Patients with a diagnosis of GIST and available biopsy and/or cytology from their primary tumor were included. All patients had a formalin‐fixed paraffin‐embedded material (FFPE) from a surgical specimen which confirmed the diagnosis and was used to compare the diagnostic accuracy of the two methods.

At the time of diagnosis, all FNA materials were obtained using a 0.6 mm needle guided by endoscopic ultrasound. The smears were air‐dried and then stained with May‐Grünwald Giemsa (MGG). The representativity of FNAs was rapidly evaluated on‐site by a cytopathologist. Biopsies were obtained either with forceps or using core‐needle biopsy, and all FFPE slides were stained with Hematoxylin–Eosin.

Clinical data and pathologists' reports were retrieved from the digital patient records. Sample representability was defined as the presence of tumor cells in the FNA or biopsy.

### 2.2 Ancillary studies

Immunohistochemistry (IHC) and immunocytochemistry (ICC) were performed as a part of the initial workup. IHC was performed on FFPE materials. ICC was performed smears or cytospin; aspirates were fixated with 4% formaldehyde followed by phosphate‐buffered saline, methanol, and acetone in that order.

Sanger sequencing for KIT and PDGFRA mutations was performed as part of the initial workup. The molecular analyses were performed on either FNA material, for example, pellet, cell suspensions, scrapings of MGG‐stained slides, fresh frozen material, or FFPE material from biopsies.

### 2.3 Statistics

A two‐sided Fisher's exact test was used to compare categorical variables. The Mann–Whitney *U* test was used to compare continuous variables. A *p* value of <0.05 was defined as statistically significant. No correction for multiple testing was performed.

## 3 RESULTS

### 3.1 Clinical data and patient demographics

A total of 347 patients had either a biopsy and/or a cytology material from their primary tumor available, and clinical data were available for all patients. Male:female ratio was 1:1, and the average age was 65 years (min–max 10–92). At the time of follow‐up, 65 patients had died (19%), whereof 17 (5%) were from GIST.

**FIGURE 1:**
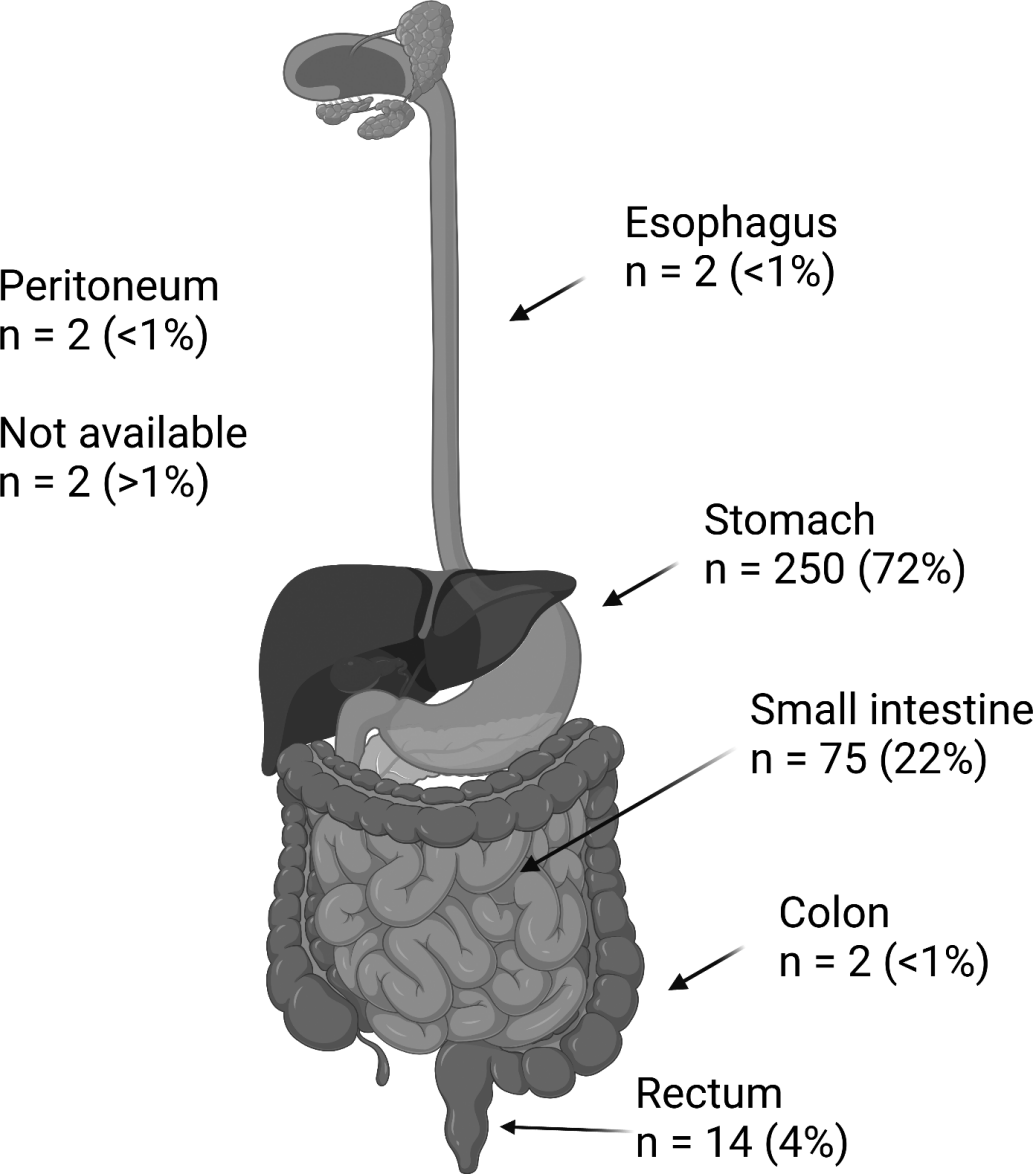
Overview of anatomical localization of gastrointestinal stromal tumors (created with biorender.com)

The majority of GISTs were located in the stomach (72%) but tumors were found throughout the rest of the gastrointestinal tract, although infrequent (Figure 1). The average tumor size in the surgical specimens was 5.6 cm (min–max 1.5–25). Median mitosis count per 50 HPFs on the final surgical specimen was 2 (min–max 0–120).

### 3.2 Sample representativity

A total of 460 samples consisting of 212 biopsies and 248 FNAs were available from the 347 patients. There was a statistically significant difference (*p* < 0.0001) in FNA samples being more representative (92%) than biopsies (77%) (Table 1). However, when separating the biopsies into mucosal and core‐needle biopsy sampling techniques, the mucosal biopsies proved to be significantly inferior (*p <* 0.0001), whereas core‐needle biopsies were superior (*p* < 0.03) to FNA regarding representativity.

**TABLE 1:** Comparison between biopsy and fine‐needle aspiration (FNA) based on the total number of samples taken

	Total samples *n* = 460		
	Biopsy	FNA	*p* value
Representative material			
All	163/212 (77%)	227/248 (92%)	<0.0001
Mucosal only	39/73 (53%)		<0.0001
Core biopsy only	102/104 (98%)		0.03
n/a	22/35 (63%)		
Diagnosis of gastrointestinal stromal tumor	161/76%	208/84%	0.035
Immunohisto/cytochemistry			
Yes	145/68%	191/77%	0.045
Molecular analysis			
Yes	37/17%	78/31%	0.0005
Sufficient material	37/17%	75/30%	0.55
Time to pathologist report Average (min–max)	12.3 (1–60)	4.5 (0–26)	<0.0001

### 3.3 Diagnostic accuracy

The numbers of correct primary diagnosis of GIST were significantly higher in FNAs (*p* < 0.0352) compared with biopsies (Table 1). In biopsies, the most common outcome of a nonrepresentative sample resulted in a misclassification of benign gastric mucosa or a faulty diagnosis of gastritis, often due to superficially sampled mucosal biopsies. Sample representativity was less of an issue in FNAs; the majority of cases that did not receive an initial diagnosis as GIST received differential diagnoses including mesenchymal tumor not otherwise specified (NOS) (*n* = 20), neoplasia NOS (*n* = 1), and schwannoma (*n* = 1).

Of the 347 patients, 100 had only biopsy available, 135 had only FNA available, and 112 had both biopsy and FNA available, often acquired during the same procedure. In the subgroup of patients who had both sampling techniques performed, there was a tendency for FNAs to have higher representability (10% vs. 26%) (Table 2). In this subgroup, 27/32 (84%) of patients received a correct FNA diagnosis where biopsy had failed to diagnose and, conversely, a biopsy provided a diagnosis in 22/28 (79%) cases that FNA had missed.

**TABLE 2:** Comparison between biopsy and fine‐needle aspiration (FNA) based on number of patients

	Total patients *n* = 347		
	Biopsy only *n* = 100	FNA only *n* = 135	Biopsy + FNA *n* = 112
Representative material	85/85%	130/96%	Both rep: 67/60% Only biopsy: 11/10% Only FNA: 30/26% Both not: 4/4%
Diagnosis of gastrointestinal stromal tumor	81/81%	124/92%	Both: 56/50% Only biopsy: 24/21% Only FNA: 28/25% Both not: 4/4%

In addition, FNAs had a significantly shorter time between sample acquisition and finalized pathologist's report (*p* < 0.001) compared with biopsies (Table 1).

In 26% (*n* = 56) of biopsies, an attempt was made to evaluate the number of mitosis; however, only 2% (*n* = 5) of biopsies contained sufficient material for the 50 HPF required for risk assessment.

### 3.4 IHC and ICC

A significantly (*p* < 0.0451) greater number of FNAs had relevant ICC (CD117 and/or DOG‐1) performed in comparison with biopsies, which is demonstrated in Table 1.

### 3.5 Molecular analysis

A total of 115 molecular analyses were performed. A significantly greater number (*p* < 0.0005) of molecular analyses were performed on FNAs (*n* = 78) compared with biopsies (*n* = 37).

Mutations in KIT were found in 87 tumors: 4 in exon 9, 82 in exon 11, 1 in exon 13, and 0 in exon 17. Mutations in PDGFRA were found in 5 tumors: 1 in exon 12 and 4 in exon 18. No mutations could be identified in 20 tumors despite a satisfactory amount of tumor cells and no additional sequencing were performed on these tumors on the surgical specimen. Sequencing failed in 3 FNA cases due to the suboptimal material.

## 4 DISCUSSION

Morphology, IHC/ICC, molecular analysis, radiology, and clinical data all contribute toward making a correct preoperative diagnosis and risk assessment of GISTs. In this study, we evaluated the viability of FNAs in diagnosing GISTs compared with biopsies in a cohort consisting of 347 patients.

Our data showed significantly higher sample representability in FNAs compared with biopsies; in a majority of cases, the nonrepresentative biopsies were too superficially sampled and only contained benign gastric mucosa. Core‐needle biopsies did, however, provide high sample representativity and diagnostic accuracy. The sampling procedure clearly had a significant impact, though the availability of rapid on‐site evaluation by staining the FNA smears with MGG to ensure that the sample is adequate also likely plays a pivotal role. As GISTs are submucosal tumor approaches with a needle, either core‐needle biopsy or FNA, seem to be the most successful method in acquiring a sufficient sample.

Further workup using ancillary techniques was possible in both FNAs and biopsies. Immunochemistry was performed on a significantly greater number of FNAs, also in part likely due to the lower representability of biopsy material. However, it has been shown that methanol‐based fixatives as those used in our patient cases may cause altered immunoreactivity.^19^ Molecular analyses of KIT or PDGFRA mutations could be performed on 78 FNAs versus 37 biopsies, which also was a significant difference. We found no issues with sample yield using FNA cytology, although previous studies have generally concluded a lower yield in FNAs compared with biopsy, notably core‐needle biopsies.^16^, ^17^, ^18^

Another limitation of diagnosing GISTs using FNA cytology is the lack of mitotic count. Although a high number of mitosis on a biopsy may offer valuable information, biopsies also consist of a limited amount of tissue which often widely falls short of the 5 mm^2^ that is the current standard for risk assessment. In our experience, surgical specimens often exhibit significant spatial variation in their mitotic activity, thus even larger biopsies may result in the false‐low mitotic count. According to ESMO guidelines,^20^ neoadjuvant treatment with imatinib is most often based on the need for volumetric reduction to ensure good margins or obvious high risk (size, necrosis, and metastasis) cases, and the lack of a preoperative mitotic count seldom influence clinical decision‐making. On the contrary, mutational status is the most important factor as it determines if the tumor will be sensitive to imatinib and is a feasible analysis to perform on FNA material. The major drawback of this approach is that it hampers the assessment of mitotic activity in surgical specimens and thus, risk evaluation and assessment of adjuvant imatinib or even inclusion in clinical trials.

Our data also showed a strikingly shorter timeframe between sample acquisition and the finalized pathologist's report for FNAs in comparison with biopsies. The difference may partially be due to the shorter time required to process FNA smears in comparison with FFPE material and partially due to well‐established routines in primary diagnosis with FNA and specialized cytopathologists. As there could be significant variations between individual laboratories regarding processing times and routines the difference in time to diagnosis may not be applied to other institutions.

In conclusion, we found advantages to both biopsy and FNA cytology in diagnosing GISTs. Although core‐needle biopsies were highly representative and accurate, mucosal biopsies often fell short of providing the correct diagnosis. FNA cytology had high sample representability and a shorter timeframe to finalize the pathologists report. Further workup including immunochemistry and molecular analyses were also fully viable to perform on FNAs. From a resource‐efficient perspective, there is also the ease of preparing FNA smears. In summary, FNA cytology is an accurate and time‐efficient method for preoperative diagnosis of GISTs both as a complement to biopsies and as a stand‐alone sampling method.

## CONFLICT OF INTEREST

The authors declare that there are no conflicts of interest.

## AUTHOR CONTRIBUTIONS

Yifan Zhang: conceptualization, data curation, formal analysis, writing—original draft, writing—review and editing. Sara Renberg: data curation, formal analysis, writing—review and editing. Andri Papakonstantinou: conceptualization, data curation, writing—review and editing. Felix Haglund de Flon: conceptualization, formal analysis, funding acquisition, writing—review and editing.

## ETHICS STATEMENT

The study was approved by the local ethical board (Regionala etikprövningsnämnden Stockholm, registration number 2013 1979‐31).

## PATIENT CONSENT STATEMENT

All patients had given informed consent for biobanking and the use of patient samples and clinical data.

## Data Availability

The data that support the findings of this study are available on request from the corresponding author. The data are not publicly available due to privacy or ethical restrictions.
